# Enhanced Gram-Negative Membrane Disruption and In Vivo Efficacy via Lysine-Arginine Enrichment of Opis16a

**DOI:** 10.1021/acsmedchemlett.5c00038

**Published:** 2025-05-01

**Authors:** Mandelie van der Walt, Carel B. Oosthuizen, Miruna Serian, Christian D. Lorenz, A. James Mason, Megan J. Bester, Anabella R. M. Gaspar

**Affiliations:** † Department of Biochemistry, Genetics and Microbiology, Faculty of Natural and Agricultural Sciences, 98822 University of Pretoria, Pretoria 0002, South Africa; ‡ Drug Discovery and Development Centre (H3D), 37716 University of Cape Town, Rondebosch 7701, South Africa; § Department of Physics, 4616 King’s College London, London WC2R 2LS, United Kingdom; ∥ Department of Engineering, 4616 King’s College London, London WC2R 2LS, United Kingdom; ⊥ Institute of Pharmaceutical Science, 4616 King’s College London, London SE1 9NH, United Kingdom; # Department of Anatomy, Faculty of Health Sciences, University of Pretoria, Pretoria 0002, South Africa

**Keywords:** wound infections, antimicrobial resistance, antimicrobial peptides, Gram-negative bacteria, Opis16a, Opis16aCterKR, arginine substitution, membrane permeabilization, molecular dynamics, *E. cloacae*

## Abstract

Infections complicate burn wound care, especially with the rise of antimicrobial resistance. Antimicrobial peptides (AMPs) offer the potential for advancing wound care by combating persistent infections. Opis16a, a scorpion venom-derived AMP, exhibits potent antibacterial activity by targeting Gram-negative membranes, causing rapid membrane disruption and bacterial cell death. Here, four novel Opis16a analogues were developed with improved membrane targeting and antibacterial efficacy. One analogue shows particular promise for topical application in Gram-negative burn wound infections. Enhanced peptide–lipid hydrogen bonding increases conformational stability, membrane insertion, and permeabilization rates. Substituting lysine residues in the C-terminal with arginine leads to the most consistent improvement in activity, selectivity for pathogen over HaCat cells, and stability in serum. In an in vivo *Galleria mellonella* burn wound model, a 5 mg/kg topical dose provides better protection than Opis16a against *Enterobacter cloacae* NICD 16103. These findings highlight the potential of optimized bactericidal AMPs to improve burn wound care.

## Graphical abstract

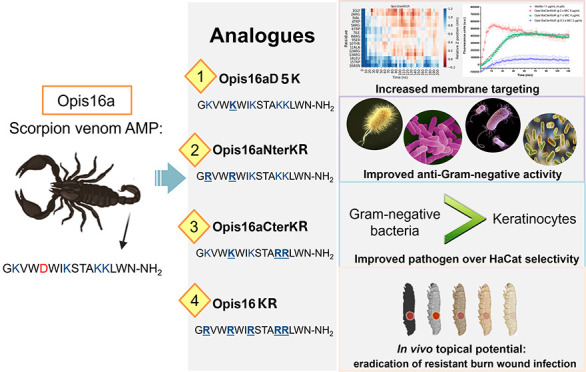

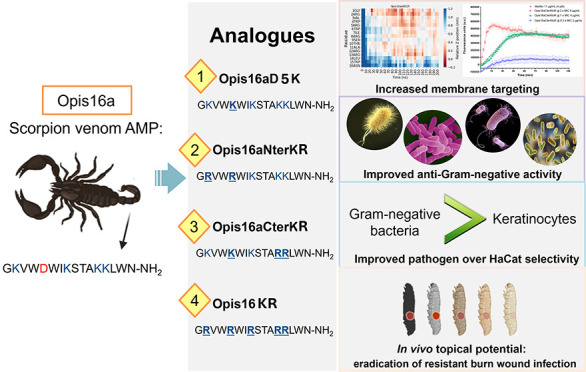

The prevention and management of wound infections are increasingly challenging due to antimicrobial resistance (AMR). An estimated 4.95 million deaths in 2019 were associated with AMR bacterial infections with infections and sepsis becoming leading causes of mortality in wound care.^1^ Burn patients are particularly vulnerable due to compromised skin barriers and immune responses.^2^ Gram-negative bacteria, such as *Enterobacter cloacae* (*E. cloacae*), dominate these infections.^3^ Indeed, studies in military hospitals revealed over 50% of severe extremity wounds contained Gram-negative organisms, with *Enterobacter spp.* causing bacteraemia in 20% of cases within 5 days.^4^ Current guidelines primarily address bacteraemia, leaving a critical gap in strategies for wound infections.^4,5^

The rise of multidrug-resistant Gram-negative infections, including carbapenem-resistant *Enterobacterales*, is a growing concern. *E. cloacae* has demonstrated increasing resistance, complicating treatment as it can develop β-lactam resistance.^4^ This gap is particularly critical in wound care, where infections involve multiple Gram-negative pathogens, leading to delayed healing, longer hospital stays, and increased mortality.^2^ In the face of such resistance, antimicrobial peptides (AMPs) offer promise as alternatives to traditional antibiotics, showing broad-spectrum efficacy and rapid action. However, while peptides, including colistin, polymyxin B, and daptomycin, are already used against resistant infections,^6,7^ application of AMPs to target Gram-negative bacteria like *E. cloacae* remains limited due to the complex outer membrane of these pathogens.^8^

Here we focus on developing novel AMPs with enhanced activity against Gram-negative bacteria by modifying Opis16a, derived from African scorpion venom, which has previously demonstrated potent antibacterial activity and low mammalian toxicity.^9^ In a burn wound infection model, Opis16a effectively treated *Acinetobacter baumannii* (*A. baumannii*) infections, comparable to polymyxin B.^9^ Here, to enhance its efficacy, we substitute aspartic acid (Asp) with lysine (Lys) or replace Lys residues with arginine (Arg), aiming to increase electrostatic attraction and membrane insertion and, consequently, improve membrane disruption. Variants included Asp5 to Lys (Opis16aD5K), N-terminal Lys to Arg (Opis16aNterKR), C-terminal Lys to Arg (Opis16aCterKR), and all Lys to Arg (Opis16aKR) (Table 1).

**Table 1:**
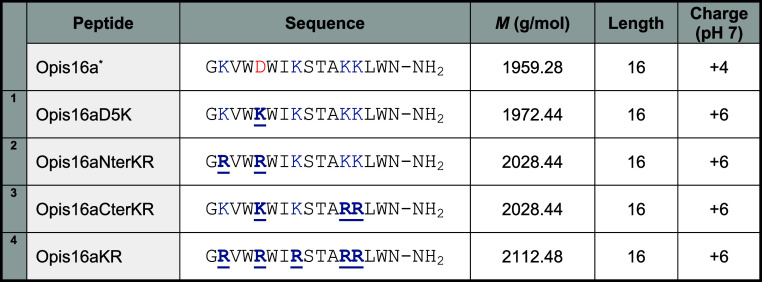
Peptide Sequences and Physicochemical Properties of Opis16a, and Its Four Analogues^a^

a Substitutions relative to Opis16a are shown in bold and underlined. Asterisk (*) indicates Van der Walt et al.^9,10^

We assessed these variants using biophysical and bacteriological methods to compare their antibacterial activity, mammalian cell toxicity, and membrane permeabilization against the parent peptide. The most promising analogue was then further evaluated for time-kill kinetics, serum stability, and protease resistance. In a burn wound *E. cloacae* infection model, this effective variant demonstrated enhanced therapeutic potential, highlighting the potential of rational modification of AMPs like Opis16a in addressing the challenges of AMR in wound care, particularly for treating Gram-negative bacterial infections.

## Opis16a Analogues Show Increased Hydrogen Bonding with Lipids

In peptide therapeutics, peptide charge plays a critical role in interactions with anionic bacterial membranes. This study investigates how substituting Asp with Lys or Arg in Opis16a affects membrane binding using MD simulations and mixed zwitterionic–anionic bilayers to model Gram-negative bacterial membranes.

Basic residues Lys and Arg, with high p*K*_a_ values, carry positive charges that facilitate electrostatic interactions and hydrogen bonding with anionic membrane lipid headgroups.^11^ Compared with Opis16a, the Opis16aD5K analogue shows increased hydrogen bonding, particularly at Lys5, which forms an average of 137 hydrogen bonds during a 200 ns simulation versus 78 bonds for Asp5 in the parent peptide (Figure 1A,B and Supporting Information Figure S1). Additionally, the substitution enhances hydrogen bonding by other Lys residues in the sequence (Figure 1B), causing an overall increase in hydrogen bonding compared with that of Opis16a (Figure 1F).

**Figure 1:**
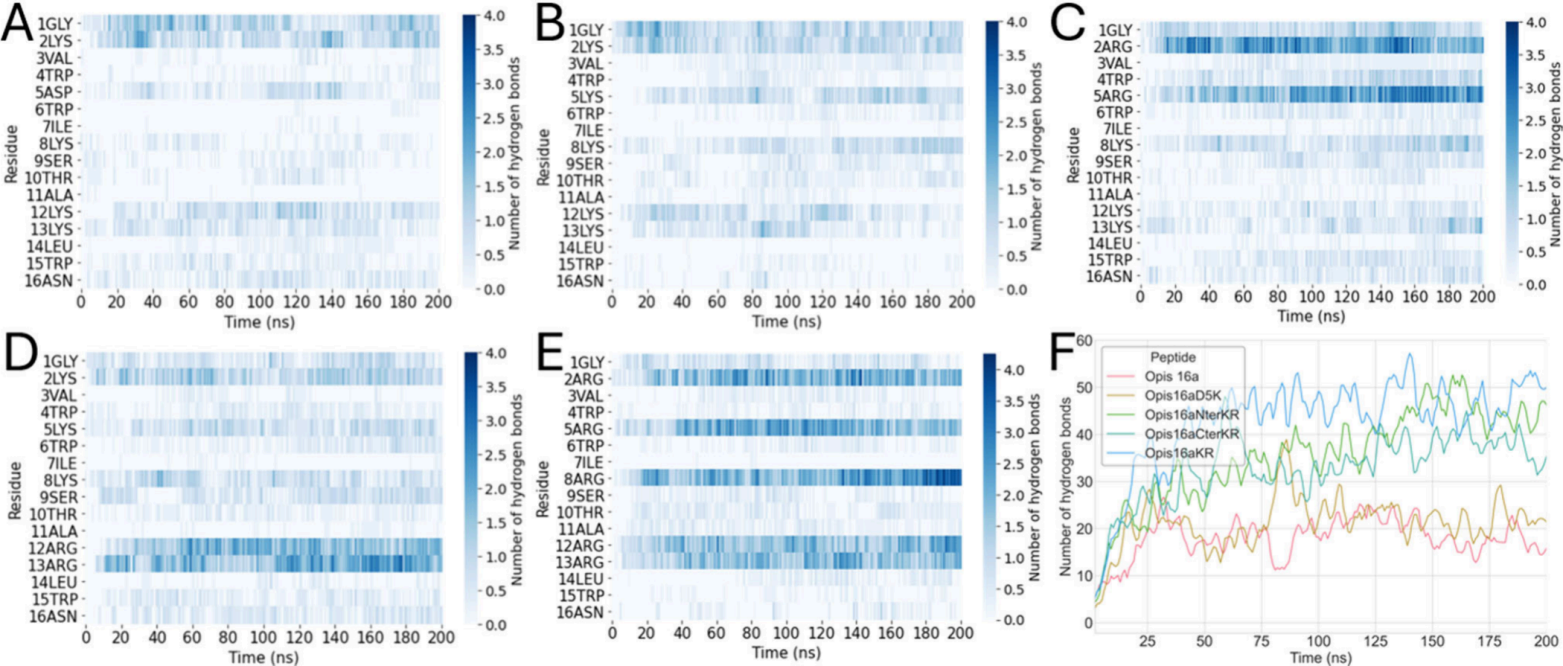
Hydrogen bonding between peptide and POPE/POPG model lipids differ among Opis16a analogues and is driven by cationic residues. Total (F) and residue-specific peptide–membrane hydrogen bonds as a function of time for Opis16a (A, red), Opis16aD5K (B, orange), Opis16aNterKR (C, lime green), Opis16aCterKR (D, dark green) and Opis16aKR (F, blue) in representative 200 ns simulations of Gram-negative model lipid bilayers.

Further improvements are observed when Lys residues at the N- or C-terminus are replaced with Arg, forming Opis16aNterKR and Opis16aCterKR, respectively (Figure 1C,D). Arg, with its guanidinium group, has more hydrogen donor atoms (five donors vs three for Lys) and forms stronger hydrogen bonds thereby increasing bacterial selectivity.^11^ Arg substitutions more than double hydrogen bonding compared to Lys, with significant contributions from residues including Arg2 and Arg5 (Opis16aNterKR) or Arg12 and Arg13 (Opis16aCterKR) (Figure 1C,D and Figure S1C,D).

Opis16aKR, with all Lys substituted with Arg, exhibits the highest hydrogen bonding along the entire sequence, not just at the termini, emphasizing the key role of Arg in enhancing peptide–membrane interactions (Figure 1E,F). Overall, the sequential enhancement in hydrogen bonding highlights the importance of cationic residues, specifically Arg, in strengthening membrane binding and antibacterial potential.

## Decreased Conformational Flexibility of the Opis16a Analogues in Model Gram-Negative Bilayers

The impact of the four modification strategies on the conformational integrity of Opis16a was assessed through steady-state CD spectroscopy and MD simulations. CD spectra were performed in Tris buffer, SDS micelles, and POPE/POPG liposomes to evaluate changes in peptide secondary structure from unstructured in the Tris environment to different structural forms depending on the complexity of the lipid environment. In both SDS and POPE/POPG the secondary structures of Opis16a and its analogues are consistent with α-helix conformation (Figure S2). Opis16a shows reduced negative band intensities in POPE/POPG, suggesting a less ordered helical structure. This could be due to the increased mobility and heterogeneity of this bilayer, coupled with Opis16a’s lower positive charge, which may result in less stable peptide–membrane interactions compared to other analogues.

Of all of the analogues, Opis16aCterKR shows the least ordered secondary structure in both membrane mimicking environments.

Unlike CD spectroscopy, which reflects average conformations over extended times, MD simulations capture nanosecond-scale structural changes, providing molecular-level insights into the secondary structure and conformational flexibility of the peptides during the initial membrane interaction (Figures 2 and 3). During this phase, Opis16a primarily adopts a polyproline II (PPII) extended conformation (φ −75°, ψ 140°), with β-sheet contributions and a type I β-turn at Trp6 (Figure 2B,C). Opis16a exhibits moderate circular variance around the psi angles, indicating conformational flexibility in Gram-negative membrane-mimicking environments (Figure 3A,B).

**Figure 2:**
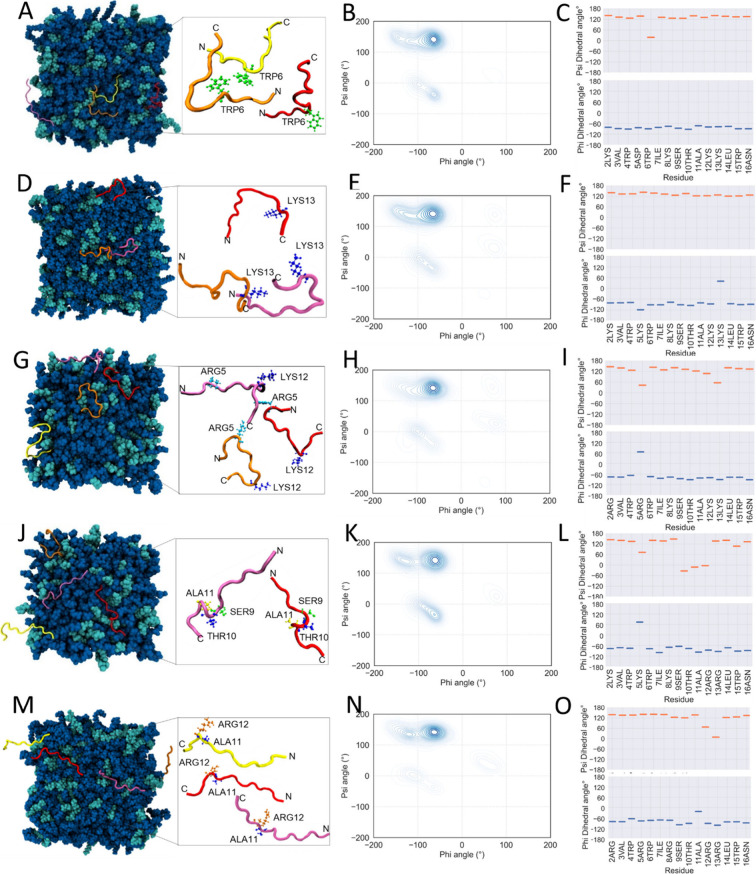
Secondary structure analysis of Opis16a and analogues in representative Gram-negative lipid bilayers. Top view snapshots showing the conformation of four peptide monomers (A, D, G, J, and M) binding to POPE/POPG bilayers in silico. Ramachandran contour plots showing the average dihedral angle concentration during the last 20 ns of 200 ns simulations (B, E, H, K, N). Psi and phi angles for each residue averaged over 200 ns of simulation and four peptides (C, F, I, L,and O). Representative data are shown for Opis16a (A–C), Opis16aD5K (D–F), Opis16aNterKR (G–I), Opis16aCterKR (J–L), and Opis16aKR (M–O).

**Figure 3:**
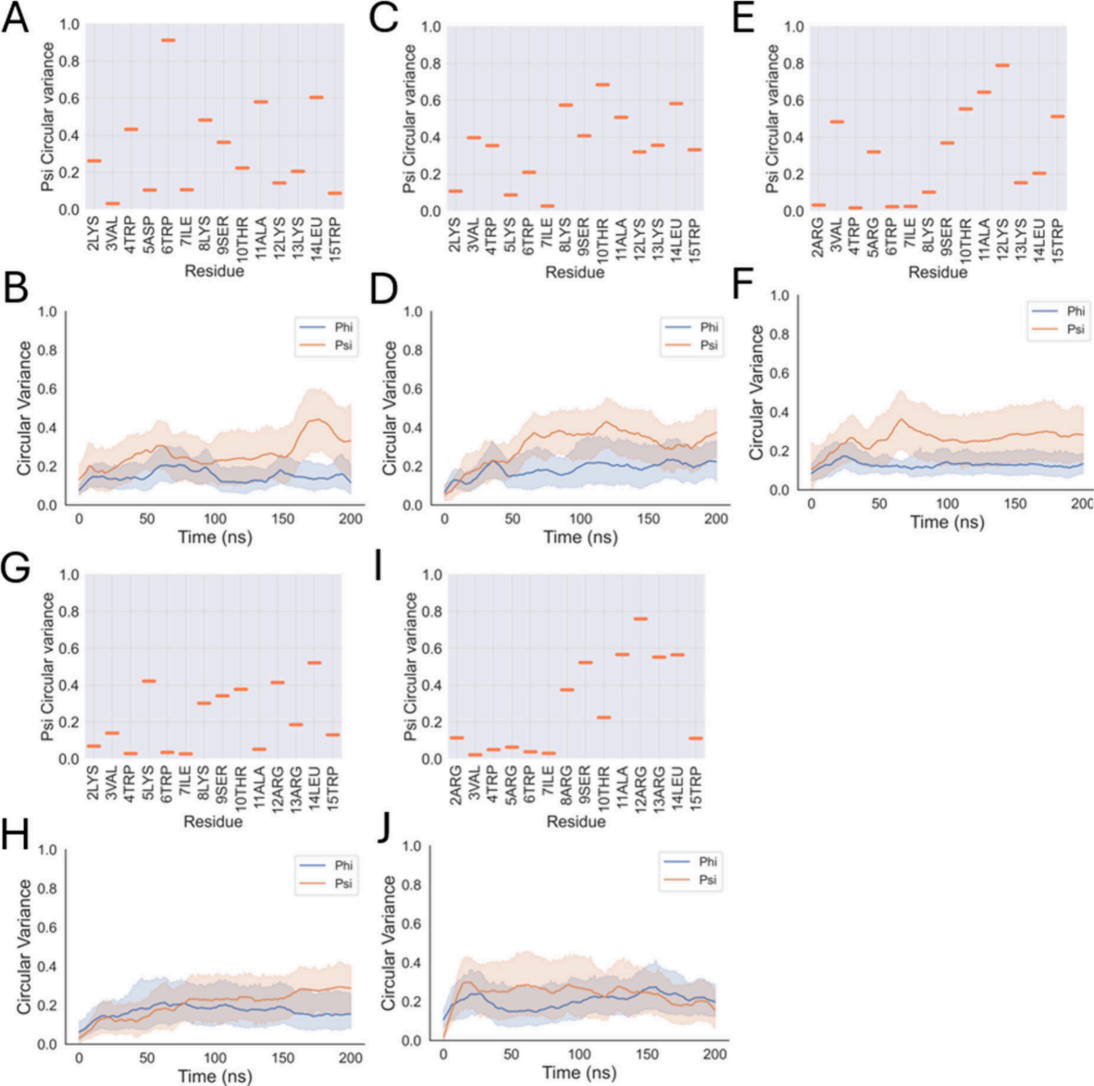
Conformational flexibility of Opis16a and analogues in representative Gram-negative bilayers. Circular variance of psi dihedral angles for each residue, averaged over 200 ns of simulation and four peptides (A, C, E, G, I), as a measure of conformational flexibility (low = rigid; high = flexible). Changes in circular variance of psi and phi angles over time, averaged for four peptides (B, D, F, H, J). Data are for Opis16a (A, B), Opis16aD5K (C, D), Opis16aNterKR (E, F), Opis16aCterKR (G, H), and Opis16aKR (I, J).

Substituting Asp5 with Lys5 (Opis16aD5K) disrupts the β-turn at Trp6 (Figure 2D), decreases the flexibility at this position (Figure 3C), and introduces turns at Lys5 and Lys13 (Figure 2D–F). These additional turns represent potential membrane interaction sites, resulting in an overall increase in conformational flexibility over time (Figure 3D).

Opis16aNterKR exhibits a conformation similar to that of Opis16aD5K, with an extended PPII structure and turns at Arg5 and Lys12 (Figure 2G–I). It shows reduced circular variance at the N-terminus compared to that of Opis16a (Figure 3E) which is likely due to stronger Arg–membrane interactions that constrain movement in this region. In contrast, the N-terminal Asp and Lys residues of Opis16a and Opis16aD5K exhibit weaker membrane interactions, resulting in greater flexibility (Figure 3A,C).

Among the analogues, Opis16aCterKR exhibits the most notable structural changes, with Lys5 inducing a turn and residues Ser9-Ala11 favoring a right-handed α-helix (Figure 2J–L). This analogue shows the lowest conformational flexibility (Figure 3H) due to the enhanced structural order and increased hydrogen bonding with the membrane (Figure 1).

Opis16aKR also adopts the extended PPII conformation, but residues 11 and 12 introduce a kink, with Arg12 favoring a type I β-turn due to the lack of steric hindrance from Ala11. This peptide also shows limited flexibility (Figure 3I,J), likely due to increased hydrogen bonding with the bilayer (Figure 1).

## Opis16a Analogues Offer Improved Antibacterial Potency

The increase in hydrogen bonding and secondary structure variation for the analogues upon interaction with model bacterial bilayers suggests altered antibacterial activity. Analogue potency was assessed against a broad-spectrum Gram-negative bacterial panel and compared with that of Opis16a (Table 2). Multidrug-resistant strains like *A. baumannii* NICD 15283, *E. cloacae* NICD 16103, *A. baumannii* ATCC 19606, and *Klebsiella pneumoniae* (*K. pneumoniae*) ATCC BAA-1705 (Tables S1 and S2), as well as susceptible strains like *E. coli* ATCC 25922, *Pseudomonas aeruginosa* (*P. aeruginosa*) PAO1, and *E. cloacae* ATCC 700323 were included.

**Table 2:** Antibacterial Activity of Opis16a and Its Analogues against Gram-Negative Pathogens

	Modal MIC, μg/mL^b^ (fold improvement)				
Gram-negative isolate^a^	Opis16a^a^	Opis16aD5K	Opis16aNterKR	Opis16aCterKR	Opis16aKR
*E. coli* ATCC 25922	64	**16** (4×)	**8** (8×)	**8** (8×)	**8** (8×)
*E. coli* ATCC 700928	4	8/4 (0.5/1×)	4 (1×)	4 (1×)	4 (1×)
*P. aeruginosa* PAO1	8	8 (1×)	4 (2×)	4 (2×)	4 (2×)
*A. baumannii* ATCC 19606	8	8 **(1×)**	4 (2×)	4 (2×)	4 (2×)
*A. baumannii* NICD 15283 (C)	8	8 (1×)	8 (1×)	8 (1×)	8 (1×)
*K. pneumoniae* ATCC BAA-1705	128	**16** (8×)	**16** (8×)	**8** (16×)	**16** (8×)
*E. cloacae* ATCC 700323	64	**16** (4×)	**16** (4×)	**8** (8×)	**8** (8×)
*E. cloacae* NICD 16103 (C)	16	8 (2×)	8 (2×)	**4** (4×)	**4** (4×)

a The minimum inhibitory concentration (MIC) is the lowest concentration that resulted in pathogen growth of <0.1 above the background absorbance. Bold values indicate a ≥4-fold improvement in potency over Opis16a. Experiments performed in triplicate with each peptide tested in duplicate (*n* = 6).

b (C) Clinical isolate.

c Activity data for Opis16a obtained from van der Walt et al.^9^

All peptides demonstrate efficacy against the entire panel, including the multidrug-resistant strains. In addition to its previously reported activity against *E. coli* and *A. baumannii,* ^9^ Opis16a also shows broad-spectrum activity against *P. aeruginosa, K. pneumoniae* and *E. cloacae*, with notable effectiveness against the multidrug-resistant clinical isolate *E. cloacae* NICD 16103, achieving an MIC lower than gentamicin and comparable to Meropenem (Table S2).

The analogues show substantial improvements in antibacterial activity compared with Opis16a. The single substitution of Asp5 with Lys5 (Opis16aD5K) enhances potency by up to 8-fold against *E. coli*, *K. pneumoniae*, and *E. cloacae*, reducing MICs to 16 μg/mL or less. Replacing Lys with Arg residues (Opis16aNterKR, Opis16aCterKR, and Opis16aKR) further improved the activity, achieving up to a 16-fold improvement. Interestingly, some peptides exhibit greater activity against resistant compared with susceptible bacterial strains. This could be due to strain-specific differences, as well as the fact that resistant strains have developed modifications to antibiotic specific targets, while they are not equipped to resist the nonspecific membrane action of AMPs.

Among the analogues, Opis16aCterKR consistently outperforms Opis16aD5K and Opis16aNterKR, and it shows the greatest improvement against *K. pneumoniae* ATCC BAA-1705 (MIC 8 μg/mL). Opis16aCterKR and Opis16aKR also exhibit enhanced efficacy against the resistant *E. cloacae* NICD 16103 isolate, surpassing all tested antibiotics with an MIC of 4 μg/mL (Table 2 and Table S2). These findings highlight Opis16a analogues, especially Opis16aCterKR and Opis16aKR, as promising candidates against multidrug-resistant Gram-negative pathogens.

## Increased Bacterial Selectivity Highlights the Potential of Opis16a Analogues

Previously, we reported an HC_50_ of >256 μg/mL and LC_50_ values of 311 ± 12 μg/mL and 118 ± 8 μg/mL for Opis16a against mammalian erythrocytes, HaCat and HepG2 cells, respectively.^9,10^ Although these values are similar to some antibacterial concentrations, Opis16a demonstrates high selectivity against *E. coli*, *P. aeruginosa*, *A. baumannii*, and resistant *E. cloacae* NICD 16103, owing to its low MICs. The toxicity of the analogues was assessed against HaCat cells, which serve as a relevant model for the intended topical application of these peptides in burn wound care. While more active against bacteria, the analogues display increased mammalian cytotoxicity, with LC_50_ values ranging from 131 to 181 μg/mL (Table 3). Notably, Opis16aCterKR demonstrates significantly lower toxicity than Opis16aD5K (*p* < 0.05), Opis16aNterKR (*p* < 0.05), and Opis16aKR (*p* < 0.01), requiring concentrations above 181 μg/mL to induce toxicity (Table 3).

**Table 3:** Toxicity and Resulting Selectivity Indexes of Opis16a and Analogues toward Gram-Negative Bacteria^a^

	HaCat toxicity and selectivity index				
Isolate	Opis16a	Opis16aD5K	Opis16aNterKR	Opis16aCterKR	Opis16aKR
HaCat toxicity					
LC_50_ + SD (μg/mL)	311 ± 12	148 ± 6	138 ± 14	181 ± 8	131 ± 5
Gram-negative bacteria					
*E. coli* ATCC 25922	4.86	9.27	**17.3**	**22.6**	**16.4**
*E. coli* ATCC 700928	**77.8**	**37.1/18.5**	**34.6**	**45.2**	**32.9**
*P. aeruginosa* PAO1	**38.9**	**18.5**	**34.6**	**45.2**	**32.9**
*A. baumannii* ATCC 19606	**38.9**	**18.5**	**34.6**	**45.2**	**32.9**
*A. baumannii* NICD 15283 (C)	**38.9**	**18.5**	**17.3**	**22.6**	**16.4**
*K. pneumoniae* ATCC BAA-1705	2.43	9.27	8.64	**22.6**	8.21
*E. cloacae* ATCC 700323	4.86	9.27	8.64	**22.6**	**16.4**
*E. cloacae* NICD 16103 (C)	**19.5**	**18.5**	**17.3**	**45.2**	**32.9**

a Bold values indicate SI > 10. Three biologically independent experiments were performed in duplicate (*n* = 6).

Despite increased toxicity, the enhanced antibacterial activity of the analogues results in improved selectivity for bacteria over mammalian cells. Like Opis16a, Opis16aD5K shows SI > 10 for five of eight bacterial strains. The Arg-containing peptides—Opis16aNterKR, Opis16aCterKR, and Opis16aKR—expand this range. Opis16aNterKR achieves an SI > 10 for six strains, including *E. coli* ATCC 25922, which Opis16aD5K does not. Opis16aKR further improves selectivity, with SI > 10 for seven strains, including *E. cloacae* ATCC 700323.

Opis16aCterKR emerges as the most promising analogue, displaying selectivity for all tested bacterial strains with SI values ≥ 22.6. It is the only peptide with SI > 10 for *K. pneumoniae* ATCC BAA-1705. Additionally, Opis16aCterKR shows higher SI values than Opis16a against *P. aeruginosa* PAO1, *A. baumannii* ATCC 19606, and *E. cloacae* NICD 16103. This improved selectivity profile suggests that Opis16aCterKR is the lead candidate for further development.

## Opis16a Analogues Show Enhanced Membrane Insertion and Permeabilization

The Opis16a analogues exhibit significantly enhanced antibacterial activity and broader selectivity, suggesting distinct mechanisms of action compared with the parent peptide. Membrane penetration and permeabilization studies reveal greater POPE/POPG bilayer insertion by the analogues, primarily through the N-terminus, as shown in 200 ns MD simulations (Figure 4). Increased hydrogen bonding (Figure 1) and stronger N-terminal–bilayer interactions (Figure 4) likely limit residue mobility, which correlates with the reduced N-terminal conformational flexibility (Figure 2).

**Figure 4:**
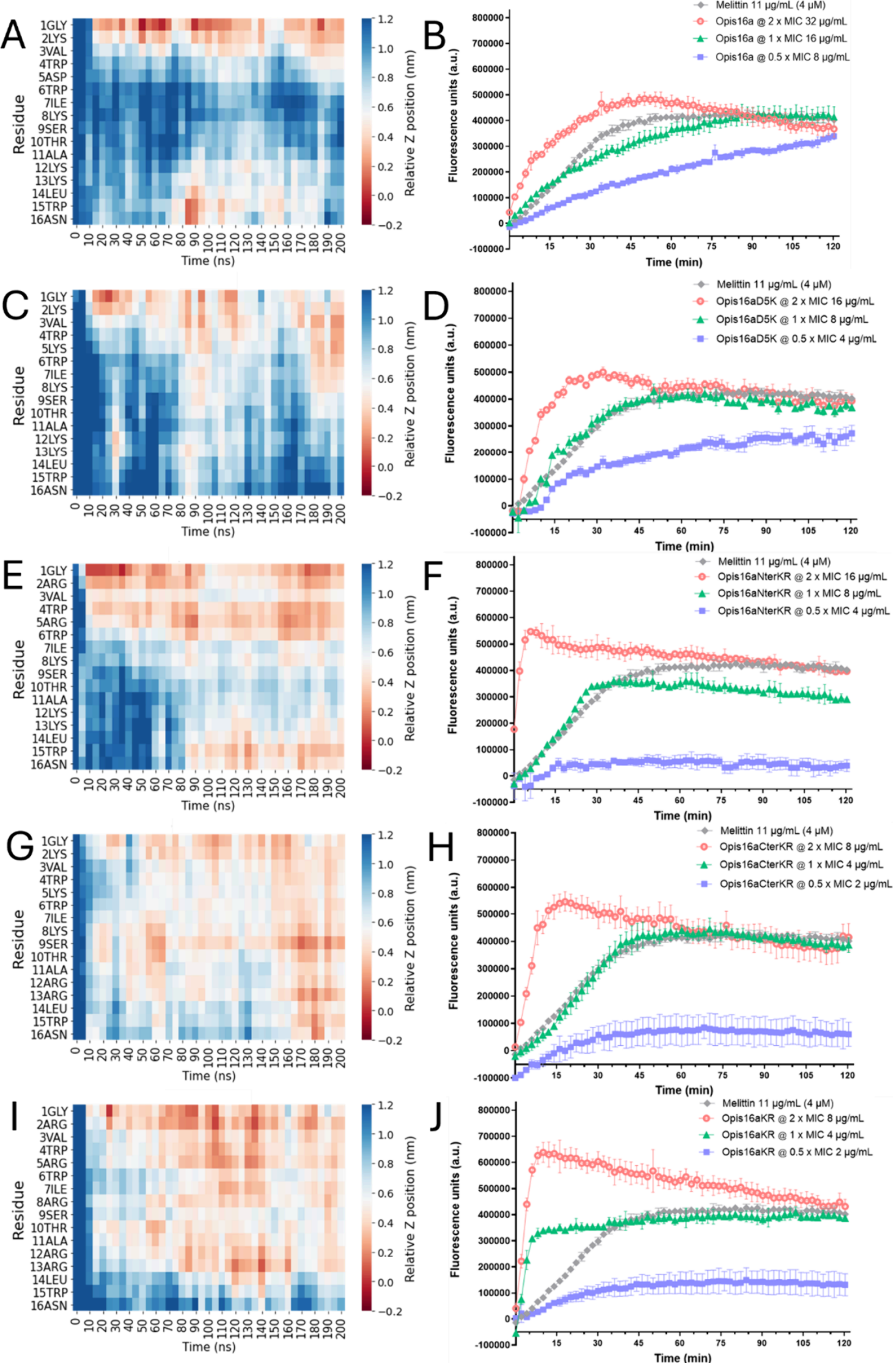
Membrane activity of Opis16a and analogues against Gram-negative bacterial membranes in silico and in vitro. (Left) Depth of Opis16a (A), Opis16aD5K (C), Opis16aNterKR (E), Opis16aCterKR (G), or Opis16aKR (I) insertion into model Gram-negative bilayers in a 200 ns simulation as the average *z*-position of each residue relative to the phosphate plane in the upper bilayer leaflet. Positive or negative values indicate the residues are above or below the phosphate group. (Right) *E. cloacae* NICD 16103 cytoplasmic membrane permeabilization by Opis16a (B), Opis16aD5K (D), Opis16aNterKR (F), Opis16aCterKR (H), and Opis16aKR (J) at 0.5×, 1×, and 2× MIC. Melittin (11 μg/mL) is used as positive control. Data show one representative experiment with mean ± SEM of a total of three biological repeats performed in triplicate.

Residue interaction with the bilayer is determined by α-carbon proximity to lipid headgroup phosphorus atoms (<6 Å).^12–14^ Opis16a inserts via its N- and C-terminal residues, with limited insertion for the midsequence due to electrostatic repulsion between anionic Asp5 and bilayer phospholipids (Figure 4A). Replacing Asp5 with Lys5 in Opis16aD5K increases the level of N-terminal insertion up to residue 8, while the level of C-terminal insertion decreases (Figure 4C). Arg-containing analogues (Opis16aNterKR, Opis16aCterKR, and Opis16aKR) demonstrate superior bilayer interaction and insertion compared to Opis16a and Opis16aD5K, as indicated by lower z-positions relative to the bilayer and shorter center-of-mass (COM) distances to the bilayer midplane (Figures 4E,G,I and 5A).

**Figure 5:**
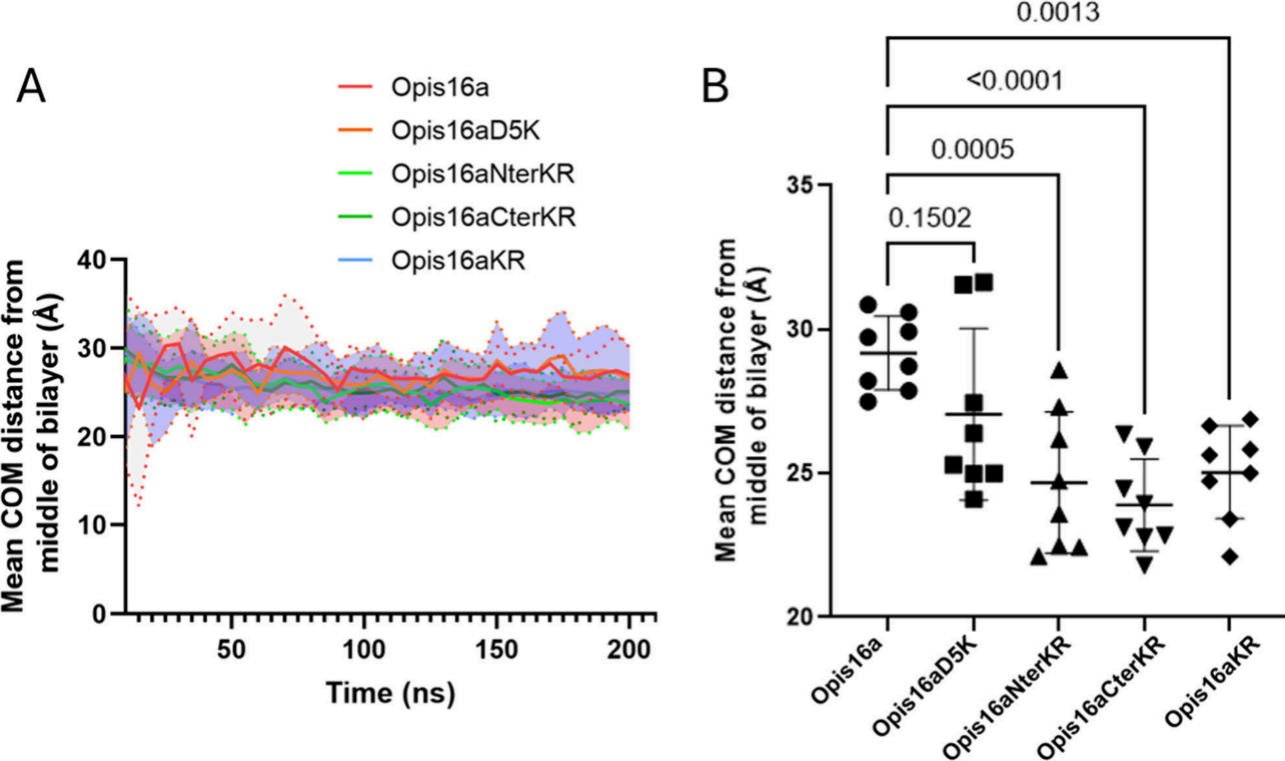
Center of mass analysis for Opis16a and analogues relative to the midplane of model Gram-negative lipid bilayers. COM distances of the peptides from the midplane of the bilayer as an average over time for two replicate 200 ns simulations (A), with the standard deviation indicated by shaded areas. Statistical significance for mean COM averaged over the last 100 ns per peptide (B) or between Opis16a and the four new analogues, determined from one-way ANOVA with Dunnett’s test.

Opis16aNterKR insertion proceeds via the first six N-terminal residues, followed by the last four C-terminal residues after 80 ns (Figure 4E). In contrast, Opis16aCterKR and Opis16aKR show bilayer interaction and insertion across the entire length of their sequences. However, Opis16aCterKR takes longer for all residues to fully insert but achieves deeper penetration once inserted (Figure 4G), while Opis16aKR inserts all residues more rapidly (Figure 4I). Among the analogues, Opis16aCterKR consistently demonstrates the deepest penetration, with significantly shorter COM distances compared to Opis16a (*p* < 0.0001) (Figure 5B).

In vitro membrane permeabilization assays confirm the enhanced membrane interaction and damage predicted in silico. All four analogues permeabilize membranes faster than Opis16a, including against the resistant clinical isolate *E. cloacae* NICD 16103 but also against *E. coli* ATCC 700928 and *A. baumannii* NICD 15283, demonstrating broad-spectrum activity (Figure S3).

At supra-MIC concentrations, the Arg-rich analogues (Opis16aNterKR, Opis16aCterKR, and Opis16aKR) exhibit the fastest permeabilization, achieving effects within 5–10 min (Figure 4F,H,J). At the MIC, Opis16a induces a steady, linear increase in *E. cloacae* NICD 16103 membrane permeabilization, peaking after 60–80 min (Figure 4B). In contrast, the analogues act significantly faster (*p* < 0.05), with Opis16aD5K reaching maximum permeabilization within 45 min (Figure 4D) and Opis16aNterKR peaking in 30 min (Figure 4F).

Although Opis16aCterKR shows slower permeabilization at early time points, it peaks within 35–40 min and causes the most extensive permeabilization, surpassing all other analogues in membrane disruption (Figures 4H and S4). Opis16aKR demonstrates the fastest effect, similar to the fastest membrane insertion observed in silico, reaching maximum permeabilization within 15 min, four times faster than Opis16a (Figure 4J).

## Opis16aCterKR Shows Rapid Killing of Resistant *E. cloacae* NICD 16103

Given the enhanced membrane disruption, superior antibacterial potency, and selectivity for bacteria over HaCat cells, Opis16aCterKR was advanced for further studies. Time-kill assays against resistant *E. cloacae* NICD 16103 show rapid bacterial eradication (Figure 6), consistent with the membrane permeabilization rates, suggesting both peptides use membrane-targeting mechanisms.

**Figure 6:**
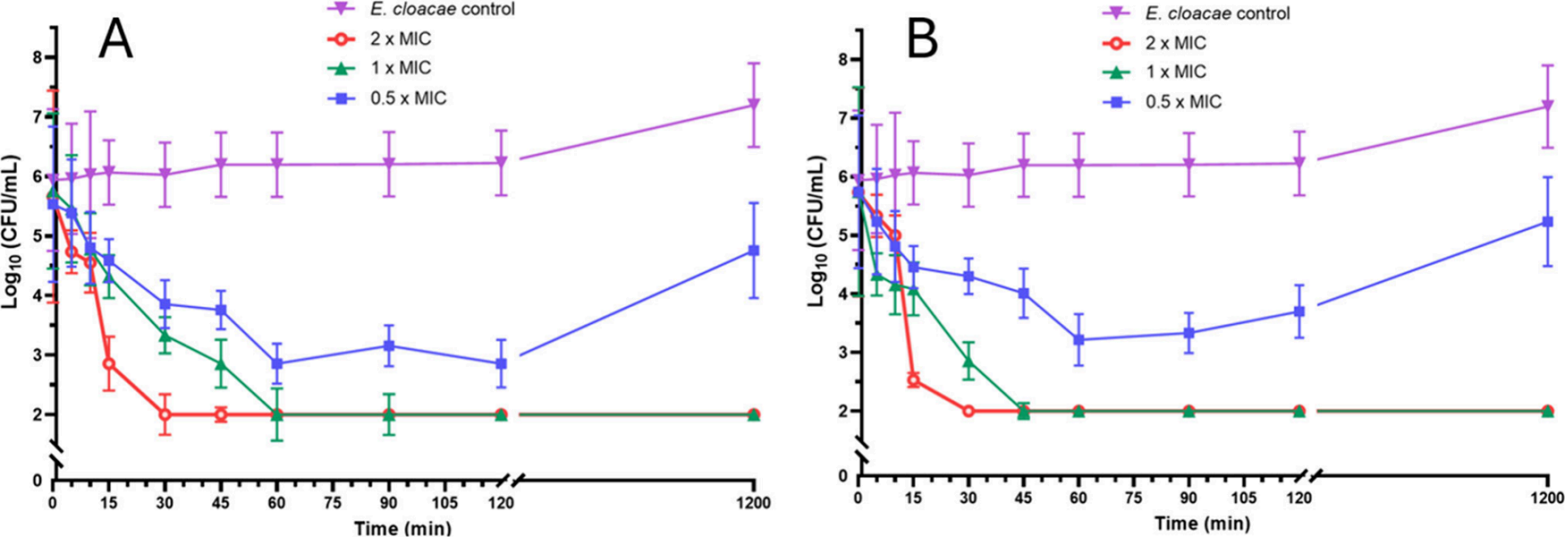
Bacterial time-kill kinetics of Opis16a compared with Opis16aCterKR. *E. cloacae* NICD 15283 treated with Opis16a (A) or Opis16aCterKR (B) at 0.5×, 1×, and 2× MIC. Data represent the mean ± SEM, for three independent experiments performed in triplicate.

Opis16a achieves complete killing at 1× MIC (16 μg/mL) within 60 min, while Opis16aCterKR requires only 4 μg/mL to achieve the same effect in 45 min (Figure 6). At 2× and 1× MIC, Opis16aCterKR causes a 4 log­(CFU/mL) reduction within 30 and 45 min, respectively, using 4-fold lower concentrations than Opis16a. At 0.5× MIC, both peptides cause a 2 to 3 log decrease in CFU/mL within 60 min, though bacterial regrowth occurs after 20 h. The enhanced efficacy and lower required dosage of Opis16aCterKR confirm its membrane-targeting mechanism and potential for further development.

## Opis16aCterKR Retains Activity in FCS but Not in Trypsin

The stabilities of Opis16a and Opis16aCterKR were assessed in the presence of trypsin and 20% FCS. Both peptides lose activity against *E. cloacae* NICD 16103 after trypsin treatment, due to cleavage at Lys and Arg residues (Figure 7A). In FCS, Opis16a shows reduced activity with the MIC increasing from 16 to 64 μg/mL (Figure 7A). However, Opis16aCterKR retains full activity, suggesting it is less susceptible to serum protein binding, a factor that can limit the clinical potential of AMPs.^15^

**Figure 7:**
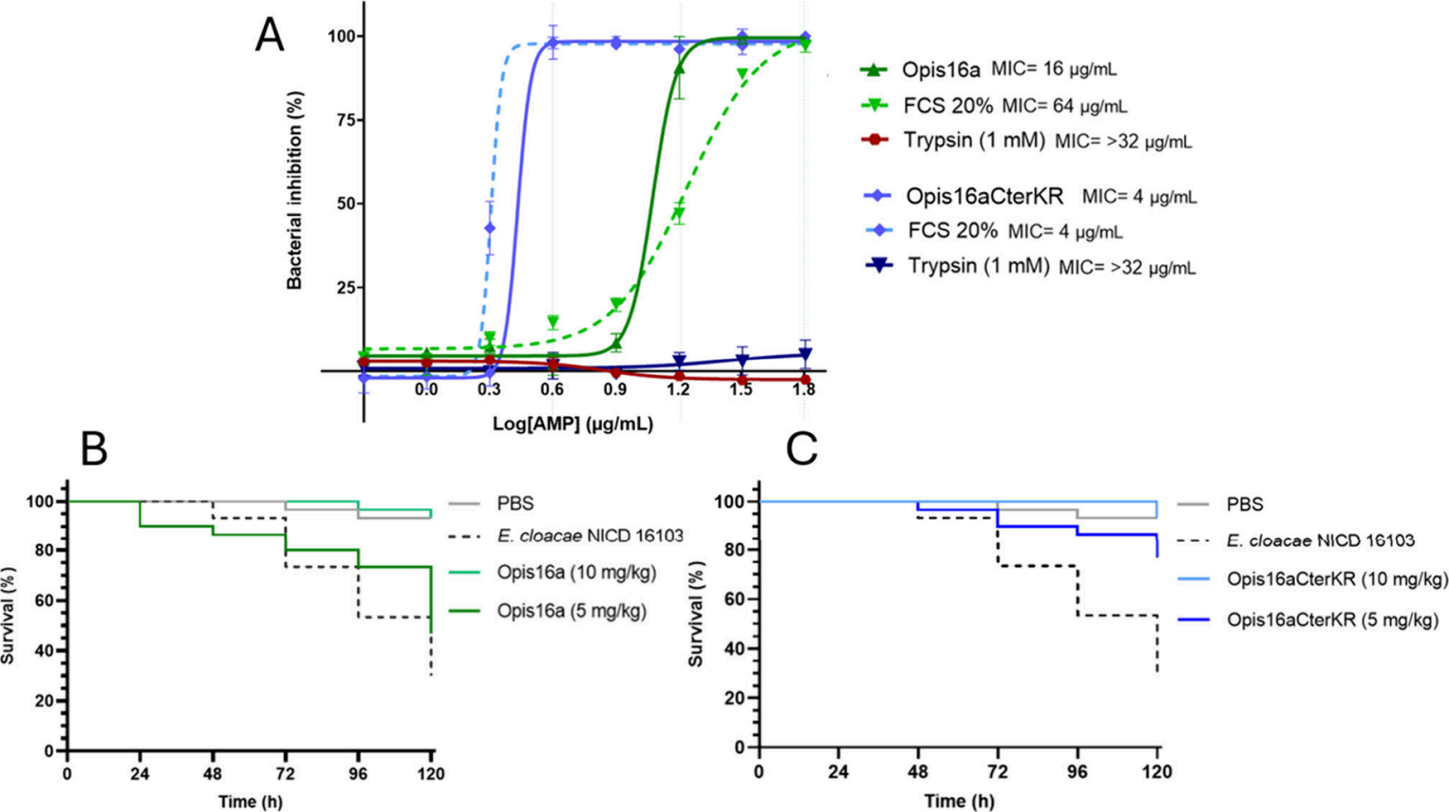
Opis16a and Opis16aCterKR remain effective against *E. cloacae* NICD 16103 in 20% serum, with Opis16aCterKR offering improved protection in a *G. mellonella* burn wound infection model. MIC testing for Opis16a and Opis16aCterKR in trypsin (0.15 U/mL) or FCS (20%) (A). Data represent the mean ± SEM, for three independent experiments performed in triplicate. Survival curves over 120 h are plotted for larvae with burn wounds only (PBS), burn and *E. cloacae* NICD 16103 infected wounds, or infected wounds treated with Opis16a (B) or Opis16aCterKR (C). Data represent three independent experiments (30 larvae per condition).

## Opis16aCterKR Shows Efficacy against In Vivo *E. cloacae* NICD 16103 Infected Burn Wounds

Opis16aCterKR demonstrates improved planktonic activity, high selectivity for *E. cloacae*, stability in 20% FCS, and potential for treating *E. cloacae* wound infections. In a *G. mellonella* wound model, *E. cloacae* NICD 16103 infection causes 70% mortality, which drops to only 7% with 10 mg/kg of Opis16a or Opis16aCterKR treatment. At 5 mg/kg, however, Opis16a shows reduced efficacy rescuing only 46% of the infected larvae (Figure 7B). In contrast, Opis16aCterKR retains significant protection, rescuing 77% (*p* < 0.001) (Figure 7C). These results highlight the therapeutic potential of Opis16aCterKR at lower doses compared to Opis16a.

## Opis16aCterKR: Novel Peptide with Considerable Potential for Future Therapeutic Development

Peptide therapeutics are gaining traction, comprising 5–6% of the global pharmaceutical market.^16^ However, of 114 AMPs approved for clinical use, only seven target wound healing or skin infections, with just two—colistin and polymyxin B—addressing Gram-negative bacteria.^17^ These AMPs are often reserved for cases of treatment failure,^17^ making Gram-negative wound infections a significant clinical challenge due to limited treatment options and increasing antibiotic resistance.

The scorpion venom-derived AMP, Opis16a, demonstrated potent efficacy against Gram-negative bacteria by rapidly disrupting bacterial membranes.^9^ Building on this, here four novel Opis16a analogues were developed, showing up to a 16-fold increase in activity and a 4-fold increase in selectivity compared with the parent peptide. The enhanced cationic nature of these analogues, achieved through substitutions of Lys or Arg, improves their membrane-targeting capabilities.

MD simulations show that the analogues form more hydrogen bonds with Gram-negative lipid bilayers than Opis16a. Although both Lys and Arg substitutions improved membrane interaction, Arg, with its guanidinium group, enables more dispersed positive charges and stronger hydrogen bonding with Gram-negative lipid bilayers compared with Lys.^11^ These interactions increase the membrane binding stability and reduce peptide flexibility, enhancing the antibacterial efficacy. While CD spectroscopy reveals similar conformations for all peptides, MD simulations provide deeper insights, showing that the analogues adopt polyproline-like conformations early in membrane interaction, as observed for other AMPs.^18,19^ The structural shifts around newly introduced cationic residues further stabilize membrane binding, as seen with increased hydrogen bonding and reduced conformational flexibility.

The role of cationic residues, particularly Arg, in the AMP efficacy is highlighted by their electrostatic interactions with bacterial membranes. As others have observed,^11^ the long, cationic side chains of Lys and Arg can facilitate multiple interactions, not only with the membrane but also with other residues within the peptide sequence, promoting the formation of more ordered structures. These residues can form noncovalent cation−π interactions with nearby tryptophan (Trp) residues,^13^ as in the case of residues 4 to 6 of the analogues, where the positive charge on the side chain of either Lys or Arg in position 5 can interact with the electron-rich indole ring of the flanking Trp residues. This interaction likely contributes to the stabilization of peptide structures such as the β-turns that are formed and may be critical for enhanced antimicrobial activity and selectivity by the Opis16a analogues.

The guanidinium group of Arg interacts more favorably with the membrane interface than Lys, promoting deeper membrane penetration and greater deformation.^11,20^ Permeabilization assays and MD simulations confirm that the Arg-containing analogues cause greater membrane damage than Opis16a. Among the variants, Opis16aCterKR shows the most significant improvement in MIC and selectivity for bacterial pathogens over mammalian HaCat cells. This analogue exhibits increased α-helix in its central sequence, deeper membrane penetration, and the lowest conformational flexibility, which may contribute to its superior efficacy.

Opis16aCterKR demonstrates broad-spectrum antibacterial activity against Gram-negative bacteria at low concentrations (4–8 μg/mL). It is particularly effective against resistant *E. cloacae* clinical isolates, outperforming Opis16a and conventional antibiotics. In vivo, Opis16aCterKR shows enhanced therapeutic potential in treating Gram-negative burn wound infections, requiring concentrations lower than those of the parent peptide.

This research highlights the benefit of incorporating Arg into AMP sequences to optimize membrane interactions and antibacterial efficacy. Given the prevalence of Gram-negative bacteria like *E. cloacae* in hospital-acquired infections, especially in burn wounds, Opis16aCterKR emerges as a promising therapeutic candidate against resistant Gram-negative pathogens.

The study contributes to AMP research in two significant ways: first, it confirms that incorporating Arg into peptide sequences enhances antibacterial activity and increases selectivity against Gram-negative bacteria; and second, it identifies Opis16aCterKR as a novel peptide with considerable potential for future therapeutic development, particularly for combating challenging Gram-negative pathogens such as *E. cloacae*, especially in the context of wound care.

## Supplementary Material



## Notes

Materials and methods; (Table S1) antimicrobial susceptibility profile; (Table S2) antibacterial activity against Gram-negative bacteria; (Figure S1) hydrogen bonding results; (Figure S2) peptide secondary structure analysis; (Figures S3 and S4) membrane permeabilization; (Figure S5) peptide starting structures (PDF)

## Abbreviations

AMP: antimicrobial peptide
MD: molecular dynamics
MIC: minimum inhibition concentration
LC: lethal concentration
SI: selectivity index
