# Supplementary material for: Enhanced Gram-Negative Membrane Disruption and In Vivo Efficacy via Lysine-Arginine Enrichment of Opis16a
Source: ACS Med Chem Lett. 2025 May 1;16(6):998–1007. doi: 10.1021/acsmedchemlett.5c00038 (PMC12169475; doi:10.1021/acsmedchemlett.5c00038)
Supplement: Supplementary file 1 [file ml5c00038_si_001.pdf]

Enhanced Gram-negative membrane disruption and *in vivo* efficacy via lysine-arginine enrichment of Opis16a

Mandelie van der Walt[a], Carel B. Oosthuizen[b], Miruna Serian[c], Christian D. Lorenz[d], A. James Mason[e*], Megan J. Bester[f] and Anabella R. M. Gaspar[a*]

[a] Department of Biochemistry, Genetics and Microbiology, Faculty of Natural and Agricultural Sciences, University of Pretoria, 0002, South Africa
[b] Drug Discovery and Development Centre (H3D), University of Cape Town, Rondebosch 7701, South Africa
[c] Department of Physics, King's College London, WC2R 2LS, United Kingdom
[d] Department of Engineering, King's College London, WC2R 2LS, United Kingdom
[e] Institute of Pharmaceutical Science, King's College London, SE1 9NH, United Kingdom
[f] Department of Anatomy, Faculty of Health Sciences, University of Pretoria, 0002, South Africa

Corresponding authors:

Prof A. James Mason, Institute of Pharmaceutical Science, Faculty of Life Science & Medicine, King's College London, SE1 9NH, UK, james.mason@kcl.ac.uk

Prof. A.R.M. Gaspar, Department of Biochemistry, Genetics and Microbiology, Faculty of Natural and Agricultural Sciences, University of Pretoria, anabella.gaspar@up.ac.za

SUPPLEMENTARY

MATERIALS AND METHODS

Materials. All peptides were obtained from GenScript in 1 mg aliquots at ≥95% purity and stored at -20°C.

Molecular dynamic simulations. Molecular dynamics simulations were conducted using the GROMACS 2021 software package[1] and the CHARMM36 all-atom force field[2]. First the initial structures of peptides were built using Avogadro (version 1.2.0)[3] and then inserted into a water box with neutralising ions using CHARMM-GUI Solution Builder[4]. Energy minimisation was carried out using the steepest descent algorithm until the maximum force is less than 1000.0 kJ/mol/nm (~5000 steps). Equilibration was carried out using the canonical NVT ensemble (fixed number of simulated particles (N), simulation cell volume (V) and temperature (T)) at 303.15 K with the Nose-Hoover thermostat for 125 ps with position restraints on the peptides. Hydrogen-containing bond angles were constrained with the LINCS algorithm. The peptide in water box simulations were run in the NPT ensemble for 1 µs using 2-fs intervals, with trajectories recorded every 2 ps. The final configurations of the peptides from these simulations (Suppl. Fig. 5) were then inserted above representative model Gram-negative bacterial membranes.

The representative Gram-negative bacterial membrane contained a total of 256 lipids (128 lipids per bilayer), composed of 1-palmitoyl-2-oleoyl-sn-glycero-3-phosphoethanolamine (POPE) and 1-palmitoyl-2-oleoyl-sn-glycero-3-phosphoglycerol (POPG) with a 75:25 (POPE/POPG) molar ratio as used in previous studies[12] and is referred to as a POPE/POPG bilayer. The lipid bilayers were built using the CHARMM-GUI Membrane Builder[5]. For each run four peptides were inserted at least 8 Å above the lipid bilayer in a random position and orientation, at least 20 Å apart. The system was solvated with CHARMM-modified TIP3P[6] water and NaCl was added to neutralise. Energy minimisation was carried out using the steepest descent algorithm until the maximum force is less than 1000.0 kJ/ml/nm (~3000–4000 steps). Equilibration was carried out using the canonical (NVT) and isothermal-isobaric (NPT) ensembles at 300 K with the Berendsen thermostat for 500 ps with position restraints on the peptides. Hydrogen-containing bond angles were constrained with the LINCS algorithm. Final simulations were run in the NVT and NPT ensemble using Nose-Hoover and Parrinello-Rahman with 2-fs intervals and trajectories recorded every 2 ps. Simulations were run for a total of 200 ns each and repeated twice, with peptides inserted at different positions and orientations.

Membrane interaction via hydrogen bonding: To better elucidate the degree of initial interaction between the peptides and Gram-negative membranes, the number of hydrogen bonds between each residue and the model lipid bilayer was calculated. The HydrogenBondAnalysis MDAnalysis module [7,8] was used to identify the presence of hydrogen bonds by measuring the distance and bond-angle between possible hydrogen bond donors and acceptors. The donor-acceptor distance (rDA) must be less than the cutoff distance of 3 Å and the donor-hydrogen-acceptor angle (θDHA) must be greater than the cutoff of 150°. The amount of hydrogen bonds and the variation thereof was determined for each residue that interacts with the Gram-negative bacterial bilayer.

Membrane binding and insertion: Membrane insertion was determined by calculating the distance between the *z*-position of the peptide α-carbons (CA) relative to the average *z*-position of the phosphate groups in the upper leaflet of the lipid bilayer. The LeafletFinder MDAnalysis module[8] was used to determine the average z-position of the phosphorous atoms within the upper leaflet of the bilayer. The relative *z*-position of the α-carbons in each residue

was determined at each timestep in the trajectory and averaged across four copies of peptide. The mean z-positions of the residue α-carbons were subtracted from the mean z-position of the upper leaflet phosphate atoms to determine the mean relative z-position (nm) of the α-carbons of the peptide residues from the phosphorous atoms, over the duration of the simulation. The relative z-positions of the α-carbons in the residues are presented as heatmaps. The mean centre of mass (COM) distance of the peptides from the midplane of the bilayer was determined over the course of the simulations and allows a measure of penetration over time. The mean COM was calculated from duplicate MD simulations and averaged across four peptides in each replicate (n = 8).

Dihedral angle and circular variance calculations: Dihedral angles are circular quantities and the circular mean of the psi and phi angles for each residue was calculated as described in Manzo *et al.* 2019[9].

Circular dichroism spectra. Far-UV CD spectra of the peptides (50 µM) were obtained in Tris buffer (5 mM, pH 7.4), sodium dodecyl sulphate (SDS) micelles (50 mM prepared in 5 mM Tris, pH 7.4) and in the presence of small unilamellar vesicles (SUVs) using a Jasco J-815 spectropolarimeter equipped with a PTC-423S Peltier temperature controller. CD analysis in both SDS micelles and POPE/POPG liposomes can reveal how the secondary structures of the peptides change in response to lipid composition.

For the preparation of SUVs, 1-palmitoyl-2-oleoyl-sn-glycero-3-phospho-(1′-rac-glycerol) (POPG) and 1-palmitoyl-2-oleoyl-sn-glycero-3-phosphoethanolamine (POPE) purchased from Avanti Polar Lipids, Inc. (Alabaster, AL) were used without purification. The lipid powders were solubilised in chloroform and dried under rotor-evaporation. To completely remove the organic solvent, the lipid films were left overnight under vacuum and hydrated in 5 mM Tris buffer (pH 7.4). The lipid suspension was subjected to five rapid freeze–thaw cycles for further sample homogenisation. POPE/POPG (75:25, mol:mol) SUVs were obtained by for 2 × 5 min with an amplitude of six microns in the presence of ice to avoid lipid degradation. The SUVs were stored at 4 °C and used within 5 days of preparation.

CD spectra were recorded from 260 to 180 nm at a constant temperature of 296.15 K, a bandwidth of 2 nm, a step size of 1 nm and a pathlength of 2 mm. The POPE/POPG SUV suspensions at a final concentration of 5 mM were used to dissolve the peptides to give a final peptide concentration of 50 µM. The same experimental conditions were used to investigate peptide secondary structure in 5 mM Tris and 50 mM SDS micelles. For data processing, a spectrum of the peptide free Tris solution, SDS solution or lipid suspension was subtracted. Peptide structures were quantitatively and qualitatively identified by examining the CD spectra and mean residue molar ellipticities (MRME, [θ], deg.cm^2.dmol^{-1}).

***Caution!** SDS is an irritant and should be handled with appropriate personal protective equipment (PPE), including gloves and safety glasses, in a well-ventilated area or fume hood. Chloroform is a toxic and potentially carcinogenic solvent. All procedures involving chloroform should be performed in a certified chemical fume hood with appropriate PPE. Freeze–thaw cycles were carried out using appropriate cryogenic precautions. Liquid nitrogen can cause cold burns, and pressure buildup in sealed containers.*

Antibacterial activity against a panel of standard laboratory strains and clinical isolates. Antimicrobial activity was evaluated against a panel of Gram-negative bacteria maintained by H3D, University of Cape Town, consisting of six laboratory reference strains and two clinical isolates. Briefly, bacterial colonies from an LB agar plate were picked and cultured in cation-adjusted Mueller Hinton broth (MHB) at 37°C with 180 rpm shaking, overnight. Cultures were

back diluted to reach a starting optical density at 600 nm (OD_{600}) of 0.1 and then diluted 100-fold to an inoculum of approximately 1 x 10^6 CFU/mL.

The peptides were serially diluted (testing concentrations ranging from 0.125 to 64 µg/mL) in media down polypropylene 96-well plates. Inoculum was added at a 1:1 ratio resulting in a starting cell density of ~5 x 10^5 CFU/mL. Gentamicin, polymyxin B, ciprofloxacin, meropenem and melittin at a concentration range of 0.063 to 32 µg/mL, were used in the antibiogram (Suppl. Table S2). Plates were incubated at 37°C for 20 h and the OD_{600} determined. Minimum inhibition concentrations (MICs) were determined from a minimum of three biological repeats. The MIC is defined as the lowest concentration that resulted in pathogen growth of <0.1 above the background absorbance.

Caution! *All bacterial work was conducted using appropriate biosafety level 2 (BSL-2) practices. Clinical isolates and reference strains were handled in a biosafety cabinet with proper PPE to prevent exposure and contamination. Culture handling and incubation steps involved standard aseptic techniques. Antibiotics such as gentamicin, polymyxin B, ciprofloxacin, meropenem, and melittin were handled with care, as they can pose health hazards through inhalation or skin contact. All biohazardous waste, including bacterial cultures and used plates, must be autoclaved or treated with appropriate disinfectants before disposal following institutional biohazard disposal protocols to prevent environmental contamination or accidental exposure.*

***In vitro* cytotoxicity screening against epidermal HaCat cells.** The HaCat cell line (Cellonex, South Africa) was cultured in Dulbecco's Modified Eagle medium (DMEM) supplemented with 10% heat-inactivated foetal calf serum (FCS) and 1% antibiotic-antimycotic (Abs) and maintained at 5–10% CO_2, 37°C, 95% humidity.

For HaCat cytotoxicity screening, 5.56 x 10^4 cells/mL were plated in a 96-well plate. After 24 h incubation, the cells were exposed to serial dilutions of each peptide from 512 to 1 µg/mL (256 to 0.5 µM) or 0.1% Triton-X-100 as the control for 21 h. Cell viability was determined by adding 10% 3-(4,5-dimethyl-2-thiazolyl)-2,5-diphenyl-2H-tetrazolium bromide (MTT) (1 mg/mL), to each well for the remaining 3 h. After incubation the media was discarded, and the formazan crystals dissolved by adding 25% DMSO in ethanol. Cell viability was determined by measuring the OD_{570} using a microplate spectrophotometer. Percentage cell viability was calculated relative to the untreated control. The LC_{50} was defined as the concentration of AMP treatment that results in 50% lethality of HaCat cells.

Selectivity towards the various bacterial species was determined using the selectivity index (SI). The SI of each peptide was calculated as the ratio of the concentration required to cause 50% cytotoxicity in HaCat cells (LC_{50}) to the MIC per bacterial species. A higher SI value indicates greater selectivity of the peptides towards specific bacteria over mammalian cells.

Caution! *Work with the HaCat cell line should be performed in a certified Class II biosafety cabinet using sterile technique to avoid contamination and ensure researcher safety. Proper PPE must be worn. Triton X-100 is a potent detergent and irritant; it should be handled with gloves and eye protection, and spills must be cleaned immediately to avoid skin and eye contact. MTT is a tetrazolium-based dye that can be harmful if inhaled or ingested, and should be handled with caution, particularly in powdered form. DMSO can enhance skin absorption of dissolved substances and should always be handled with gloves to prevent unintended systemic exposure. All waste, including media containing cytotoxic peptides, should be treated as biohazardous and disposed of according to institutional chemical and biological waste protocols.*

Cytoplasmic membrane permeabilisation. The ability of the AMPs to permeabilise Gram-negative bacterial cytoplasmic membranes was assessed with the SYTOX Green assay.

Briefly, bacterial cultures with an OD_{600} of 0.1 were diluted 5-fold in phosphate buffered saline (PBS), before SYTOX Green (10 µM) was added at a 1:1 ratio. The suspension was transferred to the wells of a black polypropylene 96-well plate containing the respective peptides to reach final concentrations of 0.5x, 1x and 2x the MIC. Melittin, at a final concentration of 11 µg/mL (4 µM), was used as positive control. The increase in fluorescence at 37°C was measured every 2 min for 120 min at excitation/emission wavelengths of 485/535 nm.

Caution! *The SYTOX Green membrane permeabilization assay must be performed under BSL-2 conditions when handling live bacterial cultures. SYTOX Green is a membrane-impermeable nucleic acid stain that is potentially mutagenic and toxic; it should be handled with gloves and eye protection, and solutions should be prepared and disposed of in accordance with institutional hazardous chemical waste guidelines. Peptides, including melittin, can be cytotoxic and must be handled with care to avoid skin or mucosal exposure. All waste containing bacterial suspensions, dyes, and peptides should be treated as biohazardous and disposed of accordingly.*

Time-kill kinetics. The concentration- and time-dependent killing capability of parent Opis16a and the most promising analogue Opis16aCterKR was determined against the resistant clinical isolate *E. cloacae* NICD 16103 using a time-kill kinetics assay. A bacterial inoculum with an OD_{600} of 0.1 was diluted 100-fold to reach 1 x 10^6 CFU/mL as described above. Killing kinetics were evaluated at 0.5x, 1x and 2x the MIC. Bacterial suspensions were added at a 1:1 ratio and incubated at 37°C. Samples were collected aseptically, at timepoints of 0, 5, 10, 15, 30, 45, 60, 90, 120, and 1200 min, each 10-fold serially diluted in sterile ddH_2O and plated on LB agar for colony counting.

Caution! *Time-kill kinetics assays must be performed under BSL-2 conditions when handling live bacterial cultures. Peptides can be cytotoxic and must be handled with care to avoid exposure. All waste containing bacterial suspensions, and peptides should be treated as biohazardous and disposed of accordingly.*

Opis16aCterKR activity in the presence of trypsin and serum. The susceptibility of Opis16aCterKR to proteolytic degradation or the binding with serum proteins was evaluated *in vitro* using trypsin or FCS. To evaluate trypsin stability, Opis16aCterKR was serially diluted in a trypsin solution (pancreas protease) to reach a final peptide concentration range of 0.125 to 64 µg/mL (0.064–32.67 µM) to 0.15 U/mL trypsin (pH 7.4). Samples were incubated for 1 h at 37°C. Peptide alone and protease alone were used as controls. After incubation the samples were heated at 60°C for 20 min for protease inactivation.

A similar strategy was followed to determine whether FCS affects the antimicrobial activity of Opis16aCterKR due to possible serum protein binding. Serial dilutions of Opis16aCterKR in a 20% FCS solution were incubated for 1 h at 37°C. Peptide alone and FCS alone were used as controls. After Opis16aCterKR incubation with trypsin or FCS, the procedure described above for determining antimicrobial activity was followed whereby an equal volume of peptide and bacteria was added to 96-well plates and incubated for 20 h at 37°C. The MIC of Opis16aCterKR pretreated with trypsin or preincubated with FCS was compared with peptide alone.

Caution! *Trypsin is a proteolytic enzyme that can cause skin and eye irritation; it should be handled with gloves and eye protection, and care must be taken to avoid aerosol formation. Heating samples to inactivate trypsin must be done using calibrated water baths, with caution to prevent burns or pressure buildup in closed tubes. FCS is a biological material and may contain residual bioactive components. It should be treated as a potential biohazard and handled using BSL-2 procedures. All steps involving live bacterial cultures must be performed under BSL-2 conditions. Waste from these assays — including*

serum-containing solutions, enzyme-treated samples, and bacterial cultures — must be decontaminated and disposed of according to institutional biohazard and chemical waste protocols.

***In vivo* antibacterial activity of Opis16aCterKR in a *G. mellonella* burn wound infection model.** The protection afforded by Opis16aCterKR against *E. cloacae* NICD 16103 infection in burn wounds was evaluated using *G. mellonella* larvae and compared with that of Opis16a. The larvae were sourced from and maintained by the FABI Biocontrol Centre and Nursery at the University of Pretoria. A similar procedure as used by Clarke *et al.* and first introduced by Maslova *et al.,* was followed [10,11]. All assays were performed on three separate occasions using a group size of 10 larvae. Prior to the experiment, *G. mellonella* larvae with similar size and weight (200-300 mg) were surface decontaminated using a cotton swab immersed in 70% ethanol. Burn wounds were induced by heating the flat head of a nail in a blue Bunsen burner flame until red hot, cooled for 12 s, and then superficially applying it for 2 s to create a 2-mm^2 burn. The burn sites were then infected with 2-3 colonies of *E. cloacae* NICD 16103 from a fresh agar plate using an inoculating loop to topically spread the bacteria over the burn wound. Uninfected controls were treated with sterile PBS. Opis16a or Opis16aCterKR at 5 or 10 mg/kg in 5 µL was topically applied to the wound 1 h after infection. Each group of larvae was housed in separate Petri dishes within a static incubator at 37°C during the duration of the experiment. Survival was monitored every 24 h over a period of 120 h. Changes in *G. mellonella* survival were analysed by the log rank (Mantel-Cox) method and plotted as Kaplan-Meier survival curves.

Caution! *BSL-2 guidelines should be followed when handling E. cloacae NICD 16103 and G. mellonella larvae. 70% ethanol is flammable; handle with care and use in well-ventilated areas, wearing appropriate PPE. The thermal injury procedure should be done with heat-resistant gloves and careful application to avoid burns. All work with live bacteria and infected larvae should be conducted under sterile conditions, and post-experiment waste must be disposed of following institutional biohazard protocols.*

Activity data and statistical analysis. Triplicate dose response assays with three technical repeats each were performed against the target pathogens. Data analyses for all *in vitro* assays were performed using GraphPad Prism® V 7.0 software (San Diego, CA, USA). Statistical analyses were performed by determining Row totals/means with standard deviation (SD) or standard error of the mean (SEM) followed by One-way analysis of variance (ANOVA) with Dunnett's multiple/selected comparison post-test. Final MD simulation trajectories were analysed with various internal scripts written in Python and Bash using MDAnalysis[7,8] or GROMACS[1] analysis modules.

Table S1: *Antimicrobial susceptibility profile provided by the NICD*

**Clinical Isolate**	**Antimicrobials**							
	**Amikacin**	**Sulbactam**	**Ciprofloxacin**	**Gentamicin**	**Meropenem**	**Imipenem**	**Tetracycline**	**Tobramycin**
A. baumannii NICD 15283 (C)	R	I	R	R	R	R	R	R
E. cloacae NICD 16103 (C)	S	R	R	R	R	R	R	R

S - Susceptible, R - Resistant, I – Intermediate

Table S2: *Antibacterial activity of four clinically used antibacterial agents, and melittin, against Gram-negative bacterial strains*

**Isolate**	**Modal MICs in µg/mL**				
	**Polymyxin B**	**Ciprofloxacin**	**Gentamicin**	**Meropenem**	**Melittin**
Gram-negative bacteria					
E. coli ATCC 25922 (S)	0.25	-	0.5	-	-
E. coli ATCC 700928 (S)	0.5	0.25	0.5	-	4
P. aeruginosa PAO1 (S)	-	0.5	4	-	2
A. baumannii ATCC 19606 (S)	1	1	**32**	-	-
A. baumannii NICD 15283 (C)	1	1	**16/32**	**32**	4
K. pneumoniae ATCC BAA-1705 (S)	0.5	**>32**	1/2	-	-
E. cloacae ATCC 700323 (S)	0.25	0.125	2	-	-
E. cloacae NICD 16103 (C)	-	**8**	**>32**	**16**	8

Bold values indicate MICs above the 2024 EUCAST breakpoint for susceptibility.

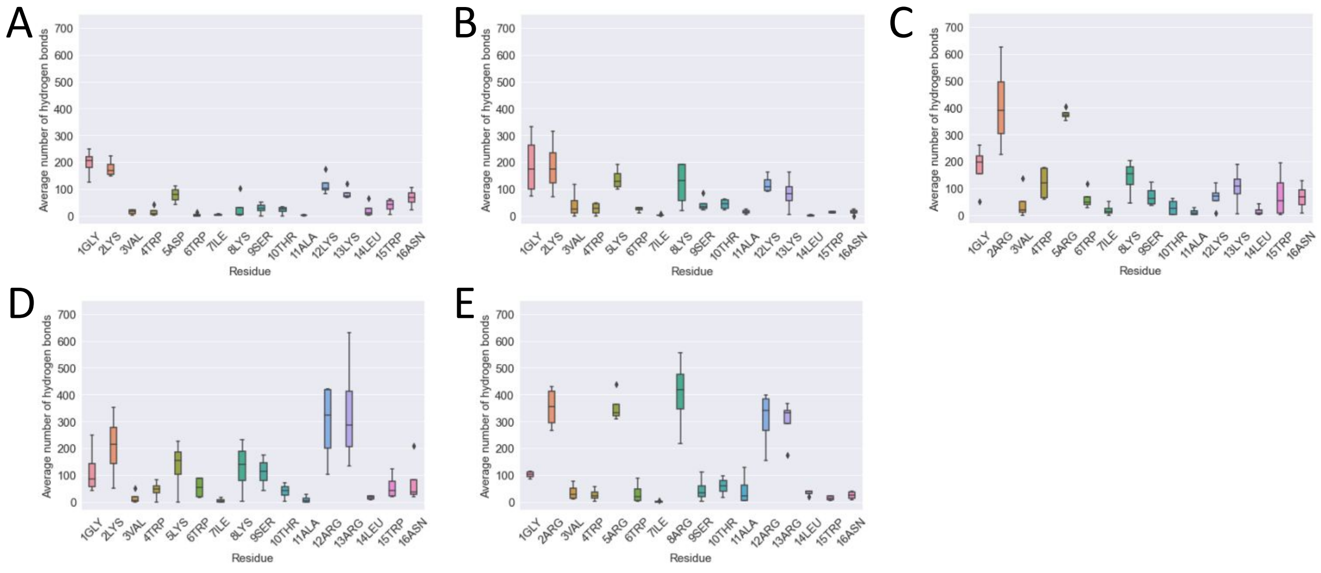

Supplementary Figure 1: Hydrogen bonding between peptide and POPE/POPG model lipids differ among Opis16a analogues and is driven by cationic residues. *Residue specific peptide-membrane hydrogen bonds are shown cumulatively for Opis16a (A), Opis16aD5K (B), Opis16aNterKR (C), Opis16aCterKR (D) and Opis16aKR (F) in representative simulations of Gram-negative model membranes.*

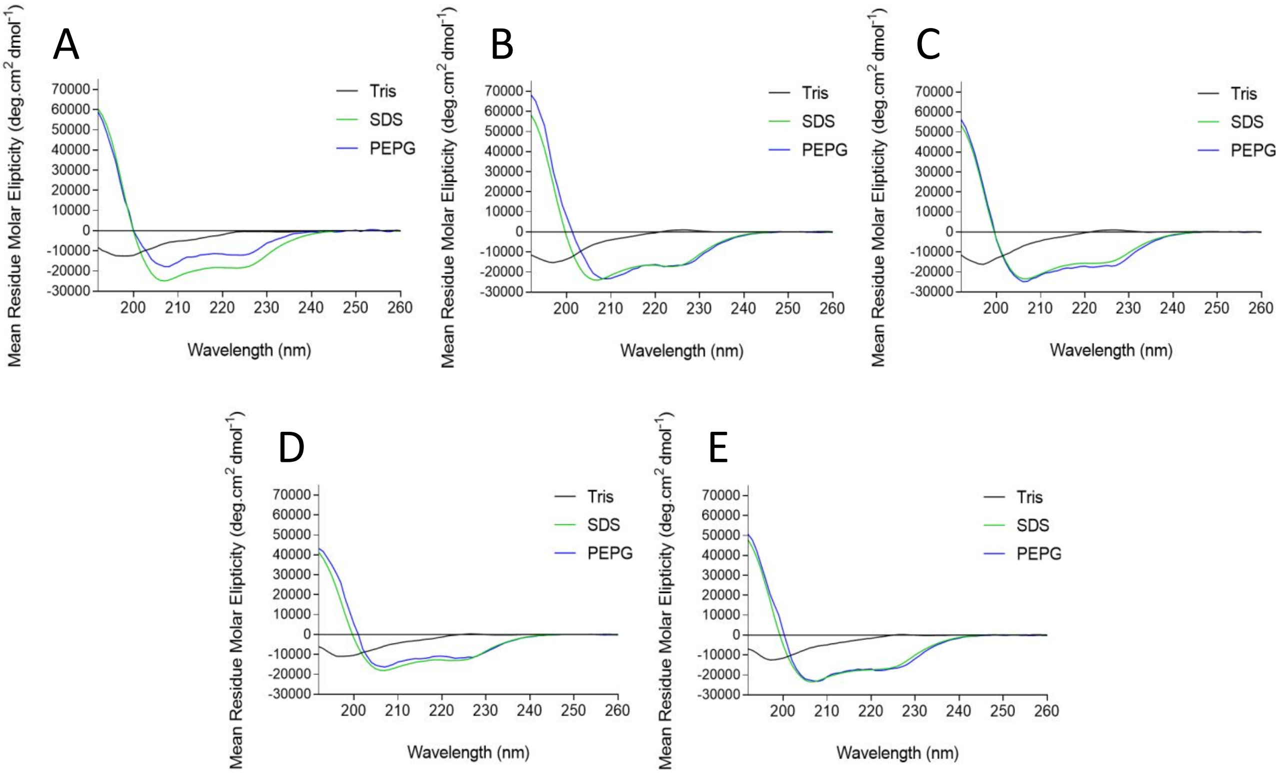

Supplementary Figure 2: Steady-state secondary structure analysis of Opis16a and analogues. *Far-UV CD spectra obtained in Tris buffer, anionic SDS detergent micelles or model Gram-negative plasma membranes comprising POPE/POPG lipids. Data are shown for Opis16a (A), Opis16aD5K (B), Opis16aNterKR (C), Opis16aCterKR (D) and Opis16aKR (E).*

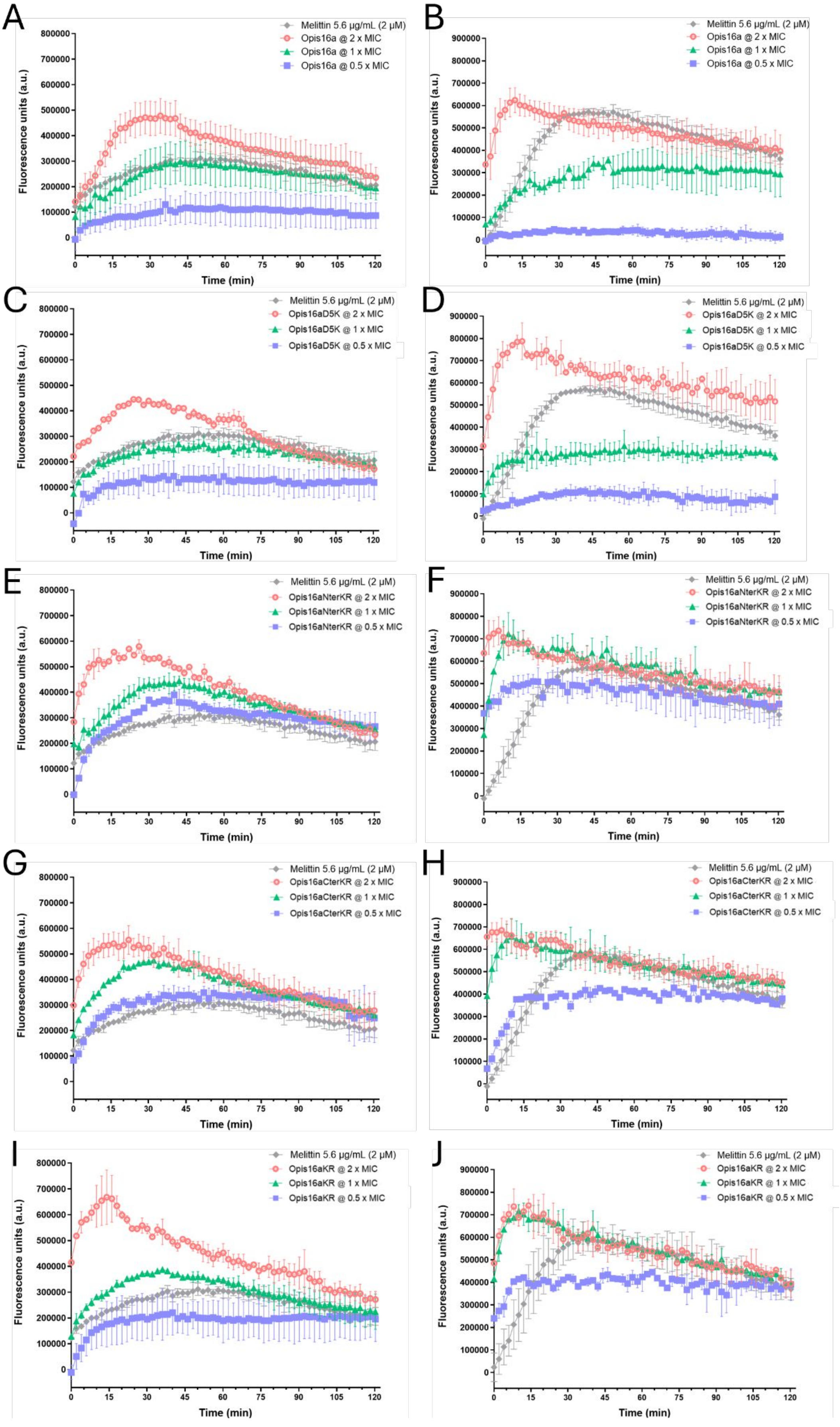

***Supplementary Figure 3: Membrane permeabilisation of Opis16a and analogues against in vitro Gram-negative bacterial membranes. (Left)** E. coli ATCC 700928 and **(Right)** A. baumannii NICD 15283 cytoplasmic*

membrane permeabilisation by Opis16a (A or B), Opis16aD5K (C or D), Opis16aNterKR (E or F), Opis16aCterKR (G or H) and Opis16aKR (I or J) at 0.5x, 1x and 2x MIC over a 2 h period. Data shows one representative experiment with mean ± SEM, of a total of 3 biological repeats performed in duplicate.

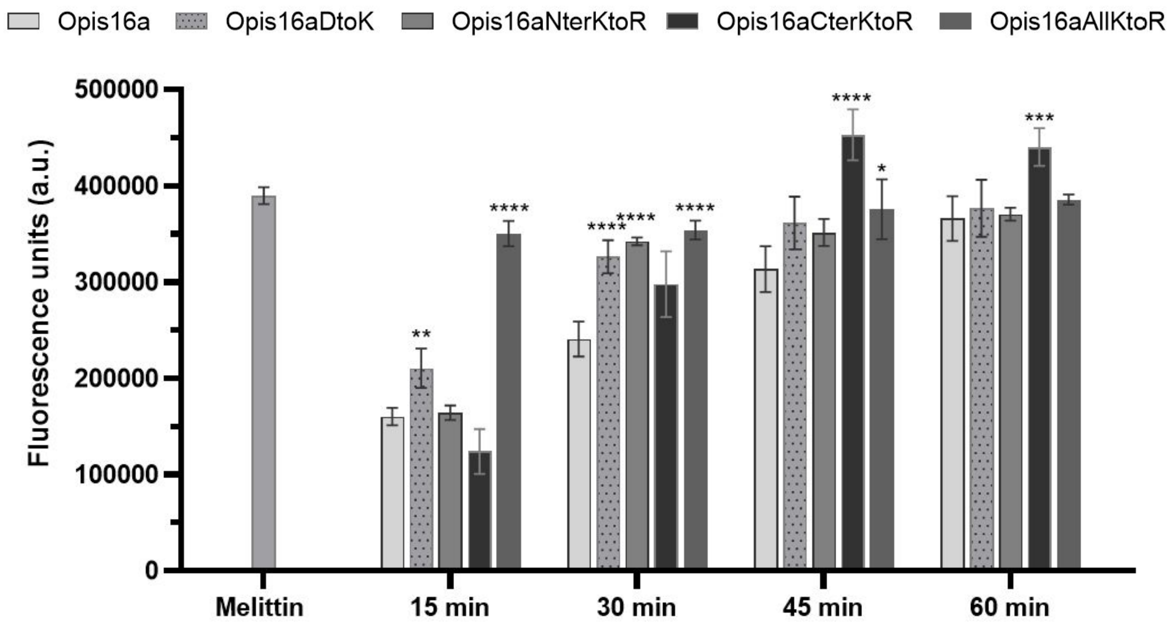

Supplementary Figure 4: Comparison of membrane permeabilisation in E. cloacae NICD 16103 induced by 1x MIC of Opis16a and its analogues at different time points. *E. cloacae NICD 16103 cytoplasmic membrane permeabilisation by Opis16a (16 µg/mL), Opis16aD5K (8 µg/mL), Opis16aNterKR (8 µg/mL), Opis16aCterKR (4 µg/mL) and Opis16aKR (4 µg/mL) at 1x MIC. Melittin at 11 µg/mL is used as positive control. Data show one representative experiment with mean ± SEM, of a total of 3 biological repeats performed in triplicate. (*: p<0.01, **: p<0.05, ***: p<0.001, ****: p<0.0001 relative to permeabilisation by Opis16a per timepoint).*

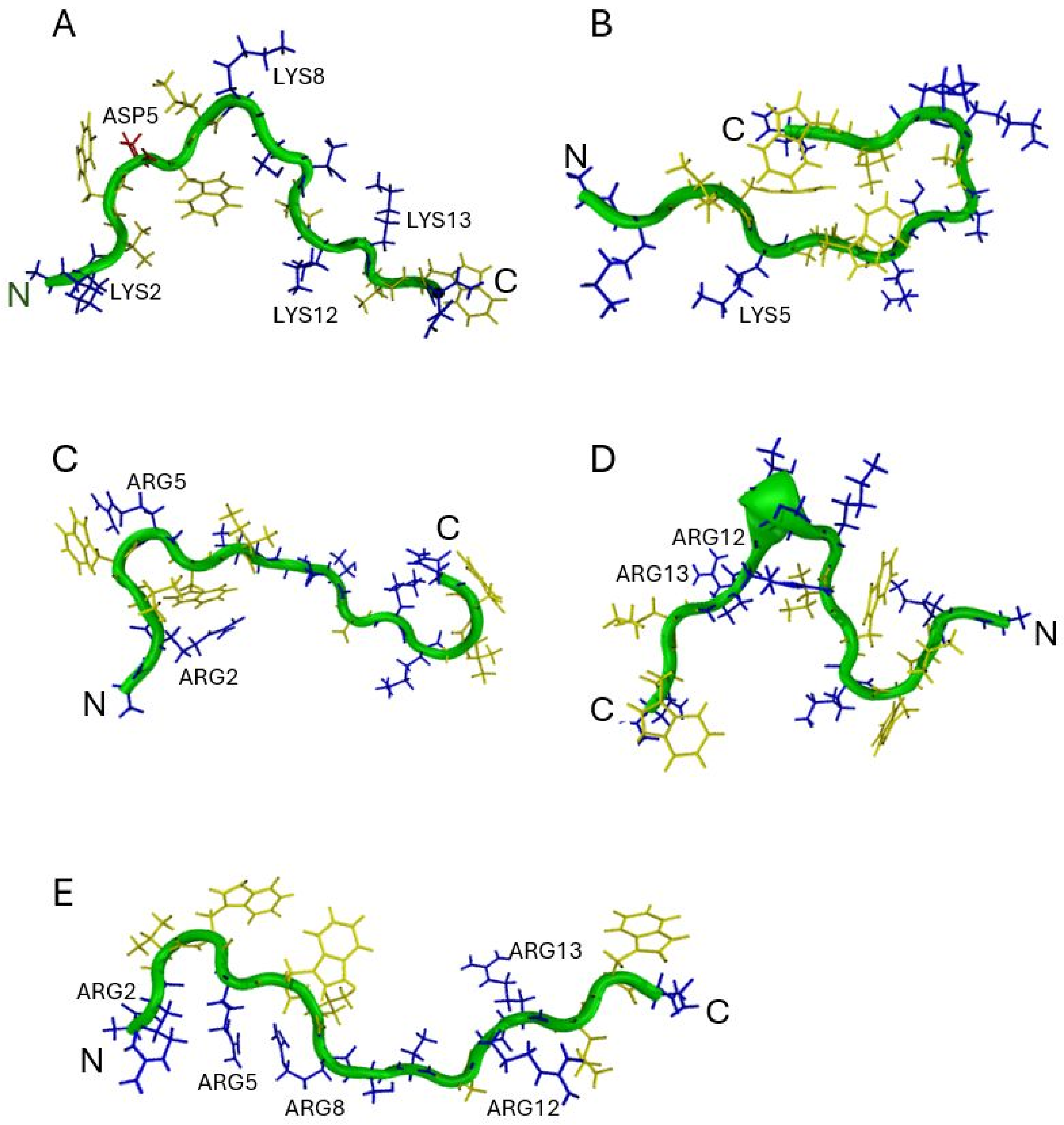

Supplementary Figure 5: Starting structures for Opis16a and its analogues for use in the MD simulations. *Opis16a (A), Opis16aD5K (B), Opis16aNterKR (C), Opis16aCterKR (D) and Opis16aKR (E). Basic residues are indicated in blue, acidic residues in red and nonpolar residues in yellow.*

Supplementary materials - References

[1] LINDAHL, E., ABRAHAM, M., HESS, B. & VAN DER SPOEL, D. (2021). GROMACS 2021 Manual (Version 2021). Zenodo. https://doi.org/10.5281/zenodo.4457591

[2] HUANG, J. & MACKERELL. J. A. D. (2013). CHARMM36 all-atom additive protein force field: Validation based on comparison to NMR data. *Computational Chemistry*, 34 (25), pg. 2135–2145. doi: 10.1002/jcc.23354

[3] AVOGADRO: An open-source molecular builder and visualization tool. Version 1.2.0. Retrieved June 2, 2024, from http://avogadro.cc/

[4] JO, S., KIM, T., IYER, V. G. & IM, W. (2008) CHARMM-GUI: A Web-based graphical user interface for CHARMM. *Journal of Computational Chemistry*, 29, pg. 1859-1865. https://doi.org/10.1002/jcc.20945

[5] WU, E. L., CHENG, X., JO, S., RUI, H., SONG, K. C., DÁVILA-CONTRERAS, E. M., QI, Y., LEE, J., MONJE-GALVAN, V., VENABLE, R. M., KLAUDA, J. B. & IM, W. (2014) CHARMM-GUI Membrane Builder toward realistic biological membrane simulations. *Journal of Computational Chemistry*, 35 (27), pg. 1997-2004. https://doi.org/10.1002/jcc.23702

[6] BOONSTRA, S., ONCK, P.R., & GIESSEN, E.V. (2016). CHARMM TIP3P water model suppresses peptide folding by solvating the unfolded state. *The Journal of Physical Chemistry B,* 120 (15), pg. 3692-3698. doi: 10.1021/acs.jpcb.6b01316.

[7] GOWERS, R. J., LINKE, M., BARNOUD, J., REDDY, T. J. E., MELO, M. N., SEYLER, S. L., DOTSON, D. L., DOMANSKI, J., BUCHOUX, S., KENNEY, I. M. & BECKSTEIN, O. (2016). MDAnalysis: A Python package for the rapid analysis of molecular dynamics simulations. In S. Benthall and S. Rostrup, editors, *Proceedings of the 15th Python in Science Conference*, pg. 98-105. Austin, TX. SciPy. doi:10.25080/majora-629e541a-00e.

[8] MICHAUD-AGRAWAL, N., DENNING, E. J., WOOLF, T. B. & BECKSTEIN, O. (2011). MDAnalysis: A toolkit for the analysis of molecular dynamics simulations. *Journal of Computational Chemistry*, 32, pg. 2319-232. doi:10.1002/jcc.21787.

[9] MANZO, G., FERGUSON, P. M., HIND, C. K., CLIFFORD, M., GUSTILO, V. B., ALI, H., BANSAL, S. S., BUI, T. T., DRAKE, A. F., ATKINSON, R. A., SUTTON, J. M., LORENZ, C. D., PHOENIX, D. A. & MASON, A. J. (2019). Temporin L and aurein 2.5 have identical conformations but subtly distinct membrane and antibacterial activities. *Scientific Reports,* 9, article 10934. https://doi.org/10.1038/s41598-019-47327-w

[10] CLARKE, M., HIND, C.K., FERGUSON, P.M., MANZO, G., MISTRY, B., YUE, B., ROMANOPULOS, J., CLIFFORD, M., BUI, T.T., DRAKE, A.F., LORENZ, C.D., SUTTON, J.M. & MASON, A.J. (2023). Synergy between Winter Flounder antimicrobial peptides. *Nature Partner Journals: Antimicrobials and Resistance*, 1 (8). https://doi.org/10.1038/s44259-023-00010-7.

[11] MASLOVA, E., SHI, Y., SJÖBERG, F., AZEVEDO, H.S., WAREHAM, D.W. & MCCARTHY, R.R. (2020). An invertebrate burn wound model that recapitulates the hallmarks of burn trauma and infection seen in mammalian models. *Frontiers in Microbiology*, 11, article 998. doi: 10.3389/fmicb.2020.00998.

[12] YUSUF, M., DESTIARANI, W., FIRDAUS, A. R. R., ROHMATULLOH, F. G., NOVIANTI, M. T., PRADINI, G. W., & DWIYANA, R. F. (2022). Residual interactions of LL-37 with POPC and POPE: POPG bilayer model studied by all-atom molecular dynamics simulation. *International Journal of Molecular Sciences,* 23 (13), article 13413. https://doi.org/10.3390/ijms231313413
